# Metaproteomics profiling of the microbial communities in fermentation starters (*Daqu*) during multi-round production of Chinese liquor

**DOI:** 10.3389/fnut.2023.1139836

**Published:** 2023-06-01

**Authors:** Jinzhi Zhao, Yi Yang, Mengjing Teng, Jianxujie Zheng, Bing Wang, Vijini Mallawaarachchi, Yu Lin, Ziyu Fang, Chengpin Shen, Shaoning Yu, Fan Yang, Liang Qiao, Li Wang

**Affiliations:** ^1^Kweichow Moutai Group, Renhuai, Guizhou, China; ^2^Department of Chemistry, Fudan University, Shanghai, China; ^3^ZJU-Hangzhou Global Scientific and Technological Innovation Center, Zhejiang University, Hangzhou, China; ^4^College of Food Science and Technology, Shanghai Ocean University, Shanghai, China; ^5^College of Engineering and Computer Science, The Australian National University, Canberra, ACT, Australia; ^6^Flinders Accelerator for Microbiome Exploration, Flinders University, Bedford Park, SA, Australia; ^7^Department of Chemistry and Chemical Biology, Rensselaer Polytechnic Institute, Troy, NY, United States; ^8^Shanghai Omicsolution Co., Ltd, Shanghai, China; ^9^Zhejiang Provincial Key Laboratory of Advanced Mass Spectrometry and Molecular Analysis, Institute of Mass Spectrometry, School of Material Science and Chemical Engineering, Ningbo University, Ningbo, Zhejiang, China

**Keywords:** *Daqu*, Chinese liquor, microbial community, quantitative metaproteomics, data-independent acquisition, fermentation, saccharification

## Abstract

**Introduction:**

The special flavor and fragrance of Chinese liquor are closely related to microorganisms in the fermentation starter *Daqu*. The changes of microbial community can affect the stability of liquor yield and quality.

**Methods:**

In this study, we used data-independent acquisition mass spectrometry (DIA-MS) for cohort study of the microbial communities of a total of 42 *Daqu* samples in six production cycles at different times of a year. The DIA MS data were searched against a protein database constructed by metagenomic sequencing.

**Results:**

The microbial composition and its changes across production cycles were revealed. Functional analysis of the differential proteins was carried out and the metabolic pathways related to the differential proteins were explored. These metabolic pathways were related to the saccharification process in liquor fermentation and the synthesis of secondary metabolites to form the unique flavor and aroma in the Chinese liquor.

**Discussion:**

We expect that the metaproteome profiling of *Daqu* from different production cycles will serve as a guide for the control of fermentation process of Chinese liquor in the future.

## 1. Introduction

Due to its distinct mellow flavor, Moutai liquor is one of the most popular Chinese liquor (1, 2). The brewing process of Moutai liquor is based on spontaneous solid-state fermentation, which is a kind of traditional brewing method. The yield and quality of liquor are highly correlated with the microorganisms involved in the fermentation process, which mainly come from the fermentation starter named *Daqu* (3, 4). The fermentation starter for Moutai liquor is a high-temperature *Daqu*, whose production includes three main processes, i.e., shaping, fermenting, and ripening (5). First, crushed raw materials, such as wheat, are mixed with 40% water and 8% prepared *Daqu*, and then the mixture is made into bricks. Next, the bricks are piled inside a warehouse and fermented at 55–60°C for 40 days. After that, the bricks are ripened for around 180 days. The bricks are then crushed and mixed into powder called prepared *Daqu* that is used directly for the brewing of Moutai liquor. The prepared *Daqu* in each production cycle is relatively stable because it is a mixture of different bricks. After fermentation, plenty of microorganisms and enzymes are produced (6, 7). Under an unique fermenting condition, a specific microbial community structure would be formed, containing bacteria, yeasts, and mycetes (7, 8). The prepared *Daqu*, which acts as both saccharifying and fermenting agents, has a direct impact on the yield and quantity of liquor (4, 9). Therefore, research on the composition and function of microflora in the prepared *Daqu* is the foundation for adequately comprehending the brewing mechanism of Chinese liquor, and may serve for the quality control of Chinese liquor production.

To date, the composition and functional activities of microorganisms in *Daqu* have been studied by various methods. Early researches used the classical microbial culture methods to identify the common microbes inside *Daqu* (10). However, these methods cannot identify unculturable microbes and fail to evaluate the microbial diversity and abundance inside *Daqu*. In the last few years, with the ability to analyze large genomes accurately and detect low-abundant genes, high-throughput sequencing has been widely used in researches of microbial community of *Daqu*, discovering the abundance changes of microbes during fermentation and the characteristics of microbial communities in various types of *Daqu* samples (2, 5, 11). Researchers have investigated the effects on the microbial community structure of *Daqu* from the production regions (12), production methods (13) and fermentation time (8, 14). Nevertheless, it is hard to distinguish viable organisms and transient DNA using the sequencing methods (15, 16). Furthermore, the genomic-based approaches can only predict the potential functions of genes rather than the actual expression of proteins (17), while proteins are the molecules conducting various biological functions during the brewing process.

Metaproteomics can make a connection between protein expression levels and specific microorganisms, thus realizing the study of the diversity, activity and function of *Daqu* microbial communities (18). Wu et al. (19) used two-dimensional polyacrylamide gel electrophoresis to analyze the microbial proteins in mature *Daqu* and identified 16 proteins. Wang et al. (20) identified 51 carbohydrate hydrolases in *Daqu* for liquor fermentation, and studied the synergistic effects of various saccharifying enzymes in *Daqu* for liquor fermentation. Due to the complexity of the raw materials and microbial composition, the deep profiling of *Daqu* metaproteome is still challenging. Recently, data-independent acquisition (DIA) mass spectrometry (MS) has shown the applicability to the analysis of complex metaproteomic samples (16, 21, 22). In particular, the latest library-free DIA data analysis methods (22–24), e.g., directDIA (23), can achieve deep proteome coverage and reproducible quantification without the need of pre-built spectral libraries (25, 26), showing great potential in metaproteome profiling of large cohorts of samples.

In this study, we used DIA-based quantitative metaproteomics to study the microbial communities of prepared *Daqu* during six production cycles at different times of a year. Using a protein database constructed by metagenomic sequencing, a total of 3,009 proteins, 7,872 peptides, and 82 microbial species were identified and quantified by DIA metaproteomics. The microbial composition and its changes across production cycles were revealed. Functional analysis of the differential proteins among production cycles was carried out and their related metabolic pathways were explored. These metabolic pathways were related to the saccharification process in liquor fermentation and the synthesis of secondary metabolites to form the unique flavor and aroma in Moutai liquor. We also analyzed the abundance of key enzymes in the process of starch and cellulose hydrolysis and identified the source microorganisms of the key enzymes. We expect that the metaproteome profiling of prepared *Daqu* in different production cycles will serve as a guide for the control of fermentation process of Chinese liquor in the future.

## 2. Materials and methods

### 2.1. Sample collection

Moutai liquor production requires raw materials to undergo multi-round of fermentation, and in each round newly prepared *Daqu* is added and mixed with sorghum (27). In this study, the prepared *Daqu* samples were collected from Kweichow Moutai Liquor Co., Ltd. (106°22′E, 27°51′N, Guizhou, China). From December 2020 to July 2021, the prepared *Daqu* in six production cycles were sampled, and 7 biological replicated samples were randomly collected in each production cycle, resulting in 42 samples collected in total (Supplementary Table 1). The collected samples were stored in dry ice and transported back to the laboratory within 24 h for storage at − 80°C.

### 2.2. DNA extraction, sequencing, and assembly

The 42 prepared *Daqu* samples were mixed to form a pooled sample. DNA was extracted from the pooled sample using HiPure Bacterial DNA Kits (Magen, Guangzhou, China) following the manufacturer’s instructions. After extraction, the quality of the DNA was analyzed by Qubit and Nanodrop (Thermo Fisher Scientific, MA, United States). Then the extracted DNA was subjected to sequencing using a NovaSeq 6,000 sequencer (Illumina Inc., CA, United States) with pair-end technology (PE 150). The DNA was firstly fragmented to a size of around 350 bp by sonication. Then, the fragments were end-repaired, A-tailed, and adaptor ligated by using the NEBNext ΜLtra DNA Library Prep Kit for Illumina (NEB, MA, United States) following the manufacturer’s instructions. PCR was applied on the DNA fragments with the length of 300–400 bp, and the amplified products were purified using an AMPure XP system (Beckman Coulter, CA, United States). Size distribution of the libraries were analyzed by 2,100 Bioanalyzer (Agilent, CA, United States). The libraries were also quantified using real-time PCR.

Raw data were filtered using FASTP (version 0.18.0) with the following standards: (1) removing reads with ≥ 50% bases having Phred quality scores ≤  20; (2) removing reads with ≥ 10% unidentified nucleotides (N); (3) removing reads aligned to the barcode adapter. The reads were assembled by MEGAHIT (version 1.2.9). MetaGeneMark (version 3.38) was used for gene identification, which output the amino acid sequences of the genes, and the amino acid sequences were used as the protein database for metaproteome analysis. The genes obtained by metagenomic sequencing were aligned by Minimap2 (version 2.17-r941) against the NCBI bacterial reference database and thereby the corresponding proteins were annotated with taxonomic information.

### 2.3. Protein sample preparation from *Daqu*

Each sample was weighed at 0.5 g and grinded twice with a mechanical grinder (JXFSTPRP-CL, Shanghai Jingxin Industrial Development Co., Ltd., Shanghai, China). After grinding twice with 5 steel balls (−50°C, 70 Hz, on 120 s, off 120 s), the powder was suspended in 1 mL borax/polyvinylpolypyrrolidone/phenol (BPP) solution (100 mM EDTA, 50 mM borax, 50 mM vitamin C, 30% sucrose, 10 mM Tris-base, 1% Triton-100, 5 mM dithiothreitol, 1% polyvinylpolypyrrolidone) and grinded twice with the mechanical grinder under the same conditions as above described. The samples were then suspended in 1 mL DNA extraction phenol reagent (Beijing Solarbio Science and Technology Co., Ltd., Beijing, China) followed by vortex for 2 min and centrifugation at 12000 *g* for 20 min at 4°C. The upper phenol phase was taken and mixed with 1 mL BPP solution under the same conditions as above to repeat the lysis step. Then, the upper phenol phase was taken and mixed with 5 mL 0.1 M ammonium acetate in methanol pre-cooled to −20°C, followed by precipitation for 4 h at −20°C. The purified proteins were centrifuged at 12000 *g* for 20 min at 4°C and washed twice with 1 mL 0.1 M ammonium acetate in methanol pre-cooled to −20°C. After drying at room temperature, the protein precipitates were dissolved in 300 μL of lysate buffer (1% sodium dodecyl sulfate and 8 M urea aqueous solution).

The protein was quantified using Pierce BCA protein assay kit (Thermo Fisher Scientific, MA, United States). For each sample, 200 μg proteins were taken, and 8 M urea solution and 20 μL 1 M triethylammonium bicarbonate buffer (TEAB) solution was added to reach a constant volume of 200 μL. Then, 4 μL 0.5 M tris-(2-carboxyethyl) phosphine hydrochloride (TCEP) solution was added for reduction. The mixture was vortexed at 600 rpm for 1 h at 37°C. After that, 18 μL 0.5 M iodoacetamide solution was added for alkylation for 45 min at 25°C in the dark. Then, 1.2 mL acetone pre-cooled to −20°C was added, followed by precipitation for 4 h at −20°C. The purified proteins were centrifuged at 15000 *g* for 15 min at 4°C and washed twice with 90% acetone pre-cooled to −20°C. After drying at room temperature, the protein precipitate was dissolved in 200 μL 0.1 M TEAB. Then, 20 μg trypsin (Beijing Wallis Technology Co., Ltd., Beijing, China) per 1 mg protein was added for digestion at 600 rpm for 16 h at 37°C. Peptides from each sample were desalted with MonoSpin C18 column (Shimadzu, Tokyo, Japan) and quantified using the Pierce quantitative colorimetric peptide assay kit (Thermo Fisher Scientific, MA, United States).

### 2.4. LC–MS/MS analysis

For metaproteomic analysis, all samples were analyzed by a nanospray Orbitrap Fusion Lumos Tribrid MS (Thermo Fisher Scientific, MA, United States) with a Nano ACQUITY UPLC system (Waters Corporation, MA, United States). For each sample, 10 μg peptides were redissolved in 30 μL solvent A (0.1% formic acid in water) spiked with 1 × iRT standard peptides (Biognosys AG, Schlieren, Switzerland). Then 1 μg of peptide sample was loaded to an C18 column (Acclaim PepMap, 75 μm × 25 cm, Thermo Fisher Scientific, MA, United States) and separated with a 60-min gradient (Supplementary Table 2), from 2 to 95% solvent B (0.1% formic acid, 20% water and 80% acetonitrile). The flow rate was maintained at 250 nL/min and the column temperature was maintained at 40°C. DIA mode was used to analyze the samples. The parameters were: method duration = 60 min, ion source type = NSI, spraying voltage positive ion = 2,200 V, spraying voltage negative ion = 2,100 V, ion transfer tube temperature = 320°C, cycle time = 3 s, MS desired minimum points across the peak = 9, using wide quad isolation = True, MS orbitrap resolution = 120,000, MS scan range (*m/z*) = 349.5–1500.5, MS maximum injection time = 50 ms, MS AGC target = 400,000, MS/MS isolation window = 1.6, maximum number of multiplexed ions = 0, CID activation time = 10 ms, activation type = HCD, collision energy = 32%, stepped collision energy = 5%, MS2 PTR reaction time = 50 ms, MS/MS orbitrap resolution = 30,000, MS/MS scan range (*m/z*) = 200–2000, MS/MS maximum injection time = 72 ms, MS/MS AGC target = 50,000. Sixty variable windows were set for MS/MS acquisition (Supplementary Table 3).

For parallel reaction monitoring (PRM) analysis, results of DIA experiment were used to select proteotypic or protein group specific peptides and to develop PRM assays by SpectroDive 11.6 (Biognosys AG, Switzerland). The inclusion list of the final optimized PRM method was present in Supplementary Data 1. Peptides of every 7 samples from the same production cycle were mixed by equal peptide quantity, resulting in six samples for PRM analysis. The peptides were re-dissolved in solvent A and analyzed by on-line nanospray LC–MS/MS on an Orbitrap Fusion lumos Tribrid MS (Thermo Fisher Scientific, MA, United States) coupled to an EASY-nanoLC 1,000 system (Thermo Fisher Scientific, MA, United States). 2 μL peptide sample was loaded onto a 25 cm analytical column (Acclaim PepMap C18, 75 μm × 25 cm) and separated with a 120 min-gradient starting at 2% solvent B followed by a stepwise increase to 35% B in 95 min, 60% B in 17 min, 100% B in 3 min and stayed there for 5 min. The column flow rate was maintained at 400 nL/min with the column temperature of 55°C. The electrospray voltage was set to 2 kV. PRM settings were as follow: Full MS scans with the mass range from m/z 450 to 1,350 were acquired with a resolution of 60,000, AGC target of 1 × 10^6^ and a maximum injection time of 50 ms. MS2 spectra were acquired with a resolution of 15,000, AGC target of 5 × 10^4^ and a maximum injection time of 80 ms.

### 2.5. MS data analysis

The DIA raw data were analyzed by Spectronaut (28) (version 15.4.210913, Biognosys AG, Schlieren, Switzerland). The data were searched by directDIA against a protein sequence database without the need of spectral libraries. The protein sequence database (1,648,851 entries) was built from metagenomic sequencing. Trypsin was used for proteolysis and the maximum number of missed cleavages was 2. Retention time prediction type was set to dynamic iRT. Data extraction was determined by Spectronaut based on the extensive mass calibration. Spectronaut will determine the ideal extraction window dynamically depending on iRT calibration and gradient stability. Q-value (FDR) cutoff on precursor and protein level was applied as 1%. Decoy generation was set to mutated. All selected precursors passing the filters were used for quantification. MS2 interference removed all interfering fragment ions except for the three least interfering ones. The average top 3 filtered peptides which passed the 1% *Q*-value cutoff were used to calculate the major group quantities. Only the leading protein (with the strongest evidence and ranked first in the result) in each protein group was taken into consideration in all the subsequent analysis.

The PRM raw data were analyzed by SpectroDive 11.6 with the default settings. SpectroDive calculated the ideal mass tolerances for data extraction and scoring based on its extensive mass calibration. *Q*-value (FDR) cutoff on precursor was applied as 1%. Peptides were manually inspected to verify the matched mass spectra and the peak integration.

### 2.6. Bioinformatic analysis

Principal component analysis (PCA) and PLS-DA were performed using MetaboAnalyst (16, 29) (version 5.0, https://www.metaboanalyst.ca/) and MetaboAnalystR (version 3.0, https://github.com/xia-lab/MetaboAnalystR). The quantified proteins were annotated with eggNOG (30) (version 4.5.1, http://eggnogdb.embl.de/). COG and EC number annotations were extracted from the eggNOG results. Statistical significance of the differences among the groups of samples was investigated by Kruskal–Wallis test (*p*-value < 0.05). To explore the patterns of microbial interactions, network analysis was carried out based on Spearman rank correlations. Genera with the relative abundance > 0.1% and appeared in at least 80% of the samples were shown as nodes. A connection stood for significant correlation (*p*-value < 0.05). Size of each node was proportional to the number of connections. The nodes were colored by genera occupancy. The thickness of edge was proportional to the value of Spearman correlation coefficients.

Data visualization was performed using R (version 4.0.4, https://www.r-project.org/), with the packages ggplot2 (version 3.3.5, https://github.com/tidyverse/ggplot2) and Venn Diagram (version 1.6.20). The interactive platform Gephi (31) (version 0.9.2, https://gephi.org/) was used to generate network diagrams.

## 3. Results

### 3.1. Metaproteomic characterization of prepared *Daqu*

**Figure 1:**
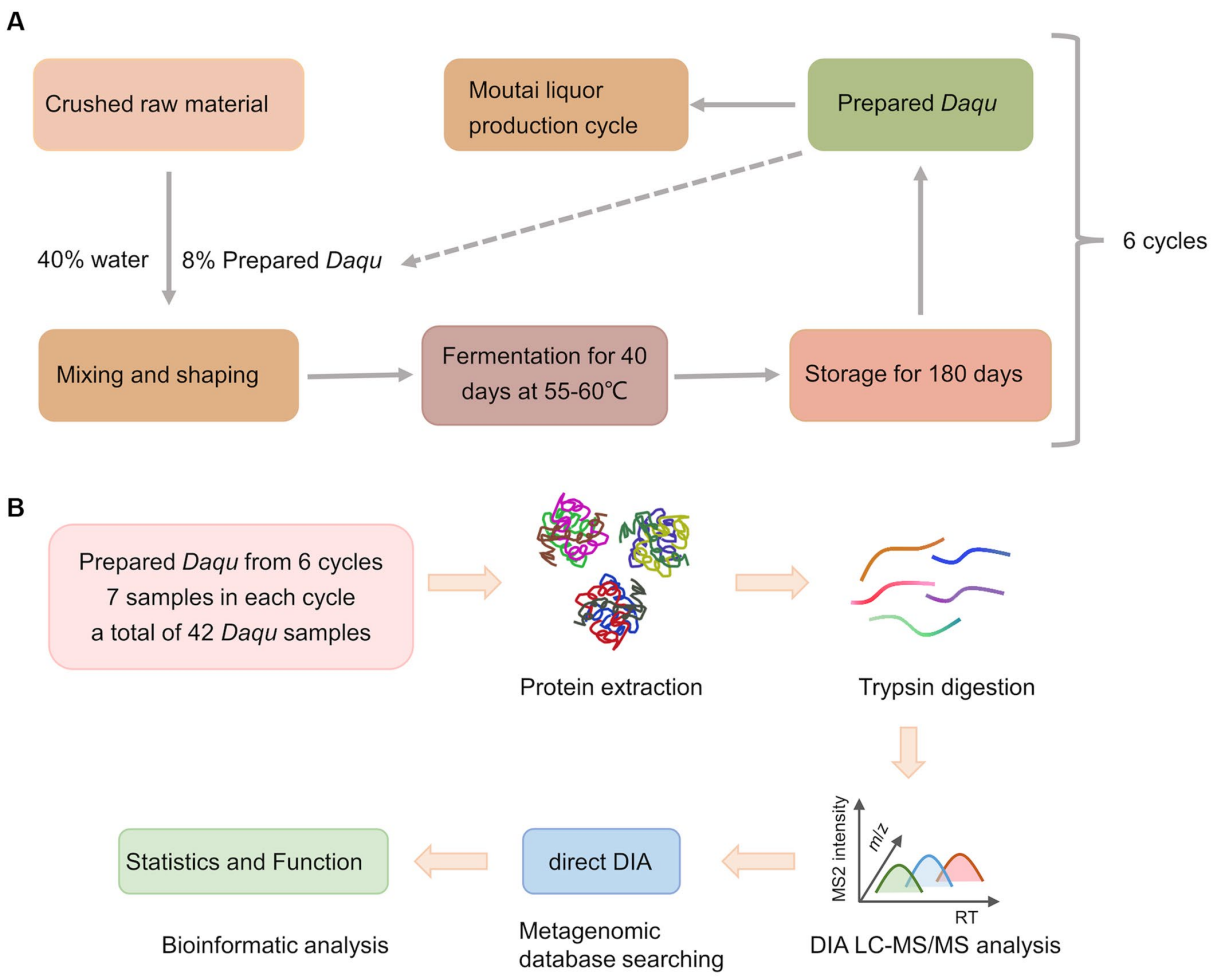
Schematic illustration of the design of this study. **(A)** The production process of Moutai liquor. **(B)** The workflow of the data-independent acquisition (DIA)-based quantitative metaproteomics analysis.

**Figure 2:**
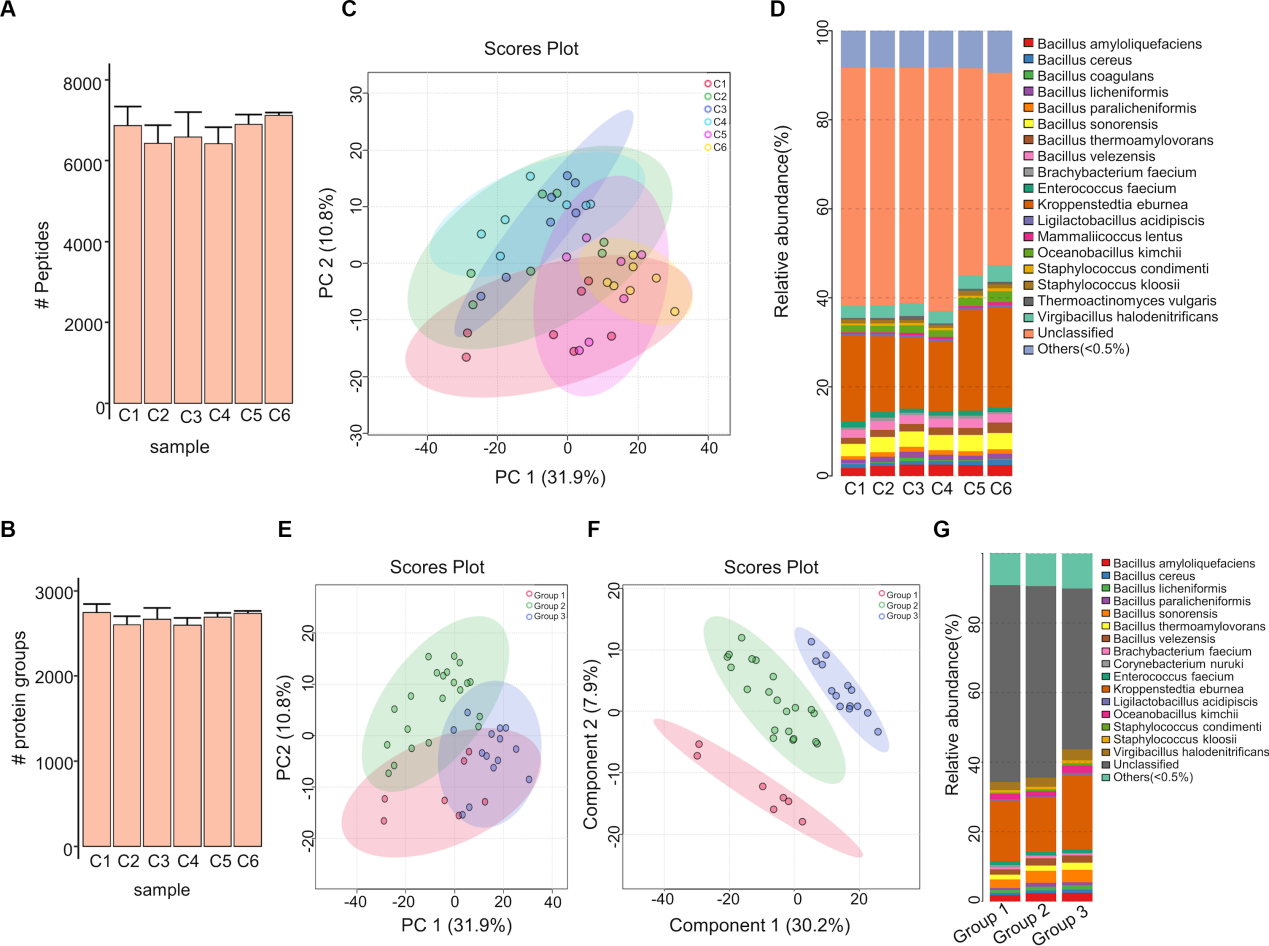
Metaproteomic characterization of the prepared *Daqu* samples. **(A,B)** The numbers of proteins **(A)** and peptides **(B)** identified and quantified from the prepared *Daqu* samples of the six production cycles (C1–C6). **(C)** PCA result of the prepared *Daqu* samples from 6 production cycles. **(D)** Relative abundance of microorganisms in the prepared *Daqu* samples from the 6 production cycles at the species level. **(E,F)** PCA **(E)** and PLS-DA **(F)** results of the 3 groups (G1–G3) of samples. **(G)** Relative abundance of microorganisms in the 3 groups of samples at the species level. G1: prepared *Daqu* sample from cycle 1; G2: prepared *Daqu* sample from cycles 2–4; G3: prepared *Daqu* sample from cycles 5–6.

The Moutai liquor production process is shown in Figure 1A. In this study, we collected a total of 42 prepared *Daqu* samples from six production cycles with seven biological replicated samples in each cycle. The prepared *Daqu* were collected from December 2020 to July 2021 (Supplementary Table 1). As shown in Figure 1B, microbial proteins were extracted from the prepared *Daqu* using mechanical grinding, and digested into peptides with trypsin. The digested samples were analyzed by label-free DIA metaproteomics. The DIA data were searched by directDIA against a sample-specific protein sequence database (1,648,851 protein entries) constructed by metagenomic sequencing of the same prepared *Daqu* samples. From the 42 samples, 3,009 proteins and 7,872 peptides were identified and quantified totally, with 2,672 ± 104 proteins and 6,720 ± 469 peptides (mean ± standard deviation) per sample (Supplementary Figure 1; Supplementary Data 2). The numbers of quantified proteins and peptides from the prepared *Daqu* samples of each production cycle were very close (Figures 2A,B). The consistent protein and peptide numbers indicated the reproducible measurements by the DIA-based metaproteomics in this study.

Based on the quantified proteins, we performed principal component analysis (PCA) to observe how the prepared *Daqu* samples in the 6 production cycles were clustered by themselves (Figure 2C). In the PCA score plot, the samples in cycles 2, 3 and 4, as well as cycles 5 and 6 were highly overlapped. We also explored the species composition of *Daqu* microbiota across the 6 production cycles (Figure 2D; Supplementary Data 3). There were in total 82 microbial species identified. Differences of microbial abundances were significant among cycle 1, cycles 2–4, and cycles 5–6. The species in *Bacillus* were less abundant in cycle 1 compared to cycles 2–4, while those in *Kroppenstedtia* and *Oceanobacillus* were more abundant in cycles 5–6 compared to cycle 1 and cycles 2–4.

According to the PCA result and taxonomic compositions, the prepared *Daqu* samples from 6 production cycles were then divided into three groups: (1) group 1: cycle 1; (2) group 2: cycles 2, 3 and 4; (3) group 3: cycles 5 and 6. Besides PCA, we performed partial least squares–discriminant analysis (PLS-DA) for the classification among the 3 groups (Figures 2E,F). The PLS-DA score plots revealed a good separation of the different groups. The species composition of *Daqu* microbiota was compared among the 3 groups as well, revealing more pronounced abundance differences than the previous 6-cycle-based comparison (Figure 2G). The quantified proteins and peptides were then counted within each group (Supplementary Figure 2). The 3 groups shared 2,930 proteins and 7,668 peptides, accounting for 97.4 and 97.4%, respectively, of all the quantified proteins and peptides from the 3 groups. All the above results indicated that the metaproteome profiles and taxonomic compositions of the prepared *Daqu* samples were relatively stable across different production cycles, while can be clustered into different groups revealing the compositional and functional changes of prepared *Daqu* during the production cycles.

### 3.2. Taxonomic compositions of the prepared *Daqu*

**Figure 3:**
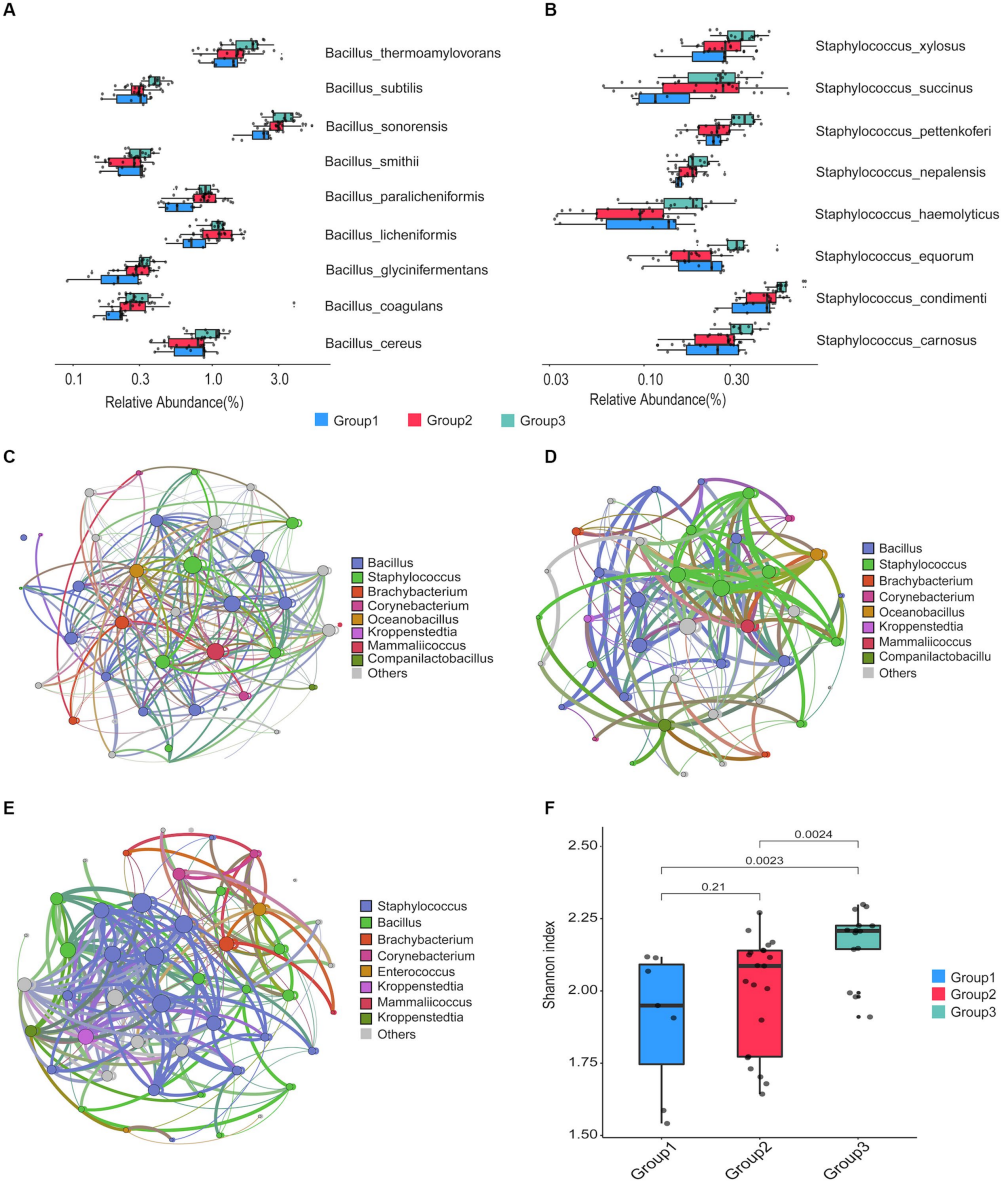
Taxonomic comparison of microbial communities among the three groups of the prepared *Daqu* samples. **(A,B)** Bar plots showing the abundance of *Bacillus* species **(A)** and *Staphylococcus* species **(B)** among the three groups. The boxes mark the first and third quantile and the lines inside the boxes mark the median; the whiskers extend from the ends of the inter-quartile range (IQR) to the furthest observations within the 1.5 times the IQR. Individual data points are overlaid as dots. **(C,D)** Co-occurrence networks of the *Daqu* microbiota from cycle 1 **(C)**, cycle 3 **(D)**, and cycle 6 **(E)**. Genera with the relative abundance > 0.1% and appeared in at least 80% of the samples are shown as nodes. A connection stands for significant correlation (*p*-value < 0.05). Size of each node is proportional to the number of connections. The nodes are colored by genera occupancy. The thickness of edge is proportional to the value of the Spearman correlation coefficients. **(F)** Box plots of alpha diversity indexes of the three groups.

From the 42 prepared *Daqu* samples, we identified a total of 18 dominant species (belonging to 10 genera) with an average relative abundance greater than 0.5% (Supplementary Data 3). The top 8 dominant species were *Kroppenstedtia eburnea* (17.83%), *Bacillus sonorensis* (3.35%), *Virgibacillus halodenitrificans* (2.81%), *Bacillus amyloliquefaciens* (2.26%), *Bacillus velezensis* (1.98%), *Oceanobacillus kimchii* (1.75%), *Bacillus thermoamylovorans* (1.69%), and *Enterococcus faecium* (1.05%). Kruskal–Wallis test was carried out to find the significantly differential species (with relative abundance > 0.15% in at least one group and FDR-corrected *p*-value < 0.05) among different groups (Figures 3A,B, as well as Supplementary Figure 3; Supplementary Data 4). The result indicates that 9 species in the *Bacillus* genus and 8 species in the *Staphylococcus* genus were more abundant in the group 3 compared to the other groups.

We next constructed a microbial community co-occurrence network for the prepared *Daqu* samples from each production cycle (Figures 3C–E; Supplementary Figure 4; Supplementary Data 5). The co-occurrence networks of microbial communities of the prepared *Daqu* samples from different production cycles showed different connectivity patterns. In the microbial community of production cycle 1, *Bacillus* was the top genera with the largest degree of connection (27.78% of the total degree of connection), followed by *Staphylococcus* (19.44%), *Brachybacterium* (5.56%), *Corynebacterium* (5.56%), and *Oceanobacillus* (2.78%). In production cycle 3, the top 4 genera with the largest degree of connection were *Bacillus* (26.83%), *Staphylococcus* (24.39%), *Brachybacterium* (2.44%), and *Corynebacterium* (2.44%). In production cycle 6, connectivity among the microbial community was primarily driven by *Staphylococcus* (31.91%), followed by *Bacillus* (23.4%) and *Brachybacterium* (4.26%). We observed higher connectivity of *Staphylococcus* in group 3, while higher connectivity of *Bacillus* in group 1 and group 2. In addition, we investigated the alpha diversity of the *Daqu* microbial communities from different production cycles by computing the Shannon diversity indexes (Figure 3F). The microbiota of group 3 exhibited significantly higher microbial diversity than the other groups.

### 3.3. Functional profiling of the differential microbial proteins

**Figure 4:**
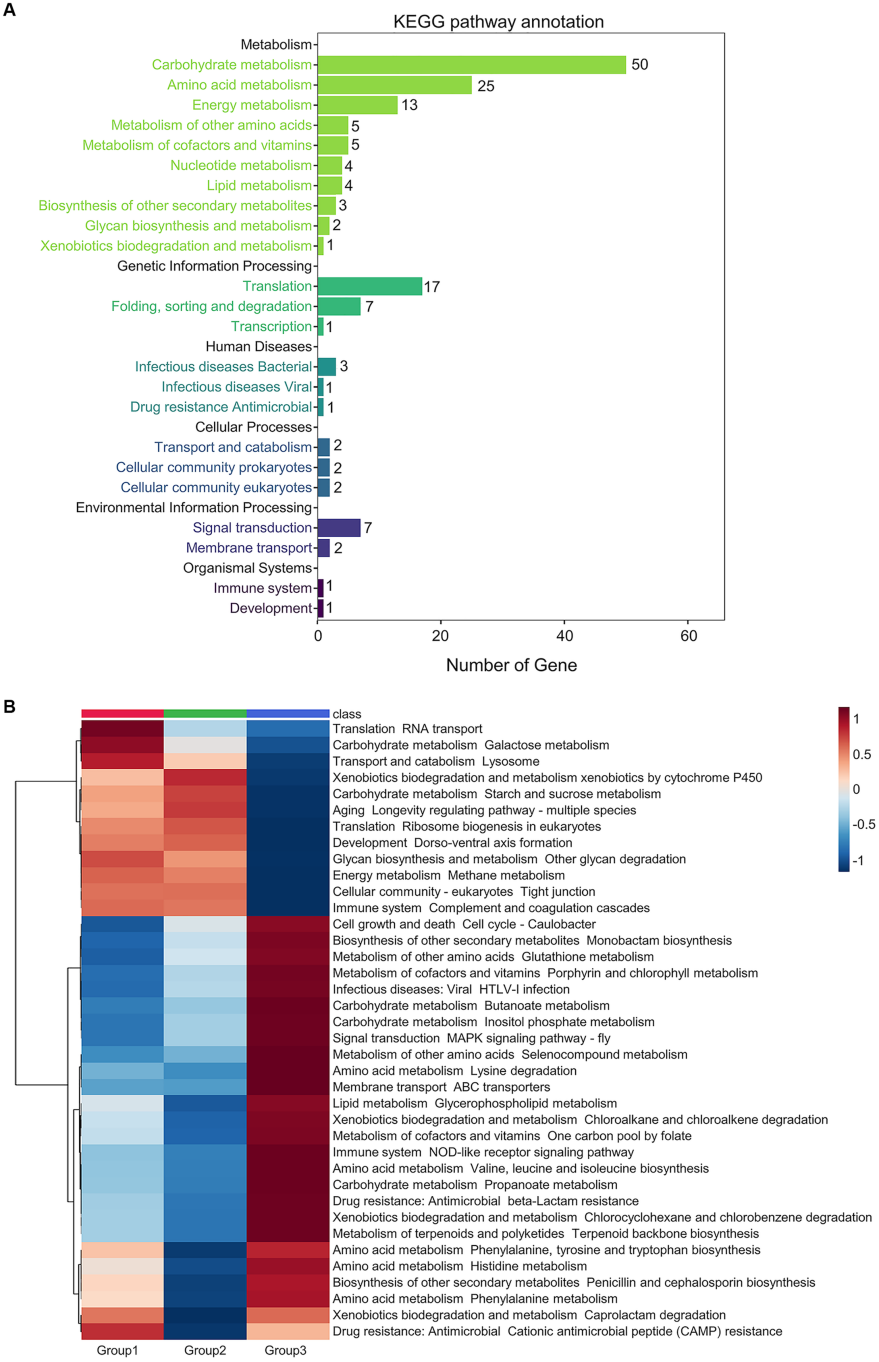
Metabolic pathways altered across the 3 groups of the prepared *Daqu* samples. **(A)** Numbers of the differential proteins (VIP score > 1.5) annotated to each pathway. **(B)** Heatmap showing relative abundance of the differential pathways (fold change > 1.25 or < 0.5, and *p*-value < 0.05 by pairwise Wilcoxon test). Abundances are normalized to *z*-scores, which are in the units of standard deviation from the mean.

A total of 306 differential microbial proteins among the three groups of prepared *Daqu* were screened by PLS-DA (VIP score > 1.5) and submitted for Kyoto Encyclopedia of Genes and Genomes (KEGG) annotation (Figure 4A; Supplementary Data 6). Among the annotated pathways, most changes occurred in the metabolism of carbohydrate, amino acid, energy, cofactors and vitamins, nucleotide, and lipid. The differential proteins also involved in the genetic information processing, including translation, folding, sorting and degradation, as well as transcription.

The abundances of the KEGG pathways were calculated based on the quantification results of all the proteins assigned to each pathway. Then, the differential pathways were screened by Kruskal-Wallis test. There were 93 differential pathways with Kruskal-Wallis *p*-value < 0.05 among the three groups of samples (Supplementary Figure 5; Supplementary Data 7), mainly focusing on amino acid metabolism (16), carbohydrate metabolism (14) and signal transduction (8). Subsequently, the above differential pathways among the three groups were pairwisely compared by Wilcoxon rank sum test, and the differential pathways with Wilcoxon *p*-value < 0.05 were extracted (Figure 4B; Supplementary Data 7). The differential pathways among all the samples of the 6 production cycles are also shown in Supplementary Figure 6. The pathways of galactose metabolism, starch and sucrose metabolism, and other glycan degradation in carbohydrate metabolism were significantly decreased in group 3 (cycles 5 and 6). The galactose metabolism pathway was more abundant in group 1 (cycle 1), while the starch and sucrose metabolism pathways were more abundant in group 2 (cycles 2–4). In contrast, amino acid metabolism, organic acid metabolism in carbohydrate metabolism, biosynthesis of secondary metabolites, and metabolism of cofactors and vitamins were significantly elevated in group 3.

### 3.4. Quantification of key enzymes in starch and cellulose hydrolysis process

Saccharification is the most basic process in the production of Chinese liquor, which involves a variety of enzymes, including amylase, glucoamylase and cellulosase. These enzymes hydrolyze starch and cellulose in the raw materials into low-molecular carbohydrates, which serve as substrates for subsequent fermentations. The quantified proteins in the 42 prepared *Daqu* samples were annotated with enzymatic function using the eggNOG database (30), and a total of 18 proteins were matched to the 4 key enzymes for starch and cellulose hydrolysis, including α-amylase, 1,4-β-fibrodiglycosidase, glucoamylase, and β-D-glucosidase.

**Figure 5:**
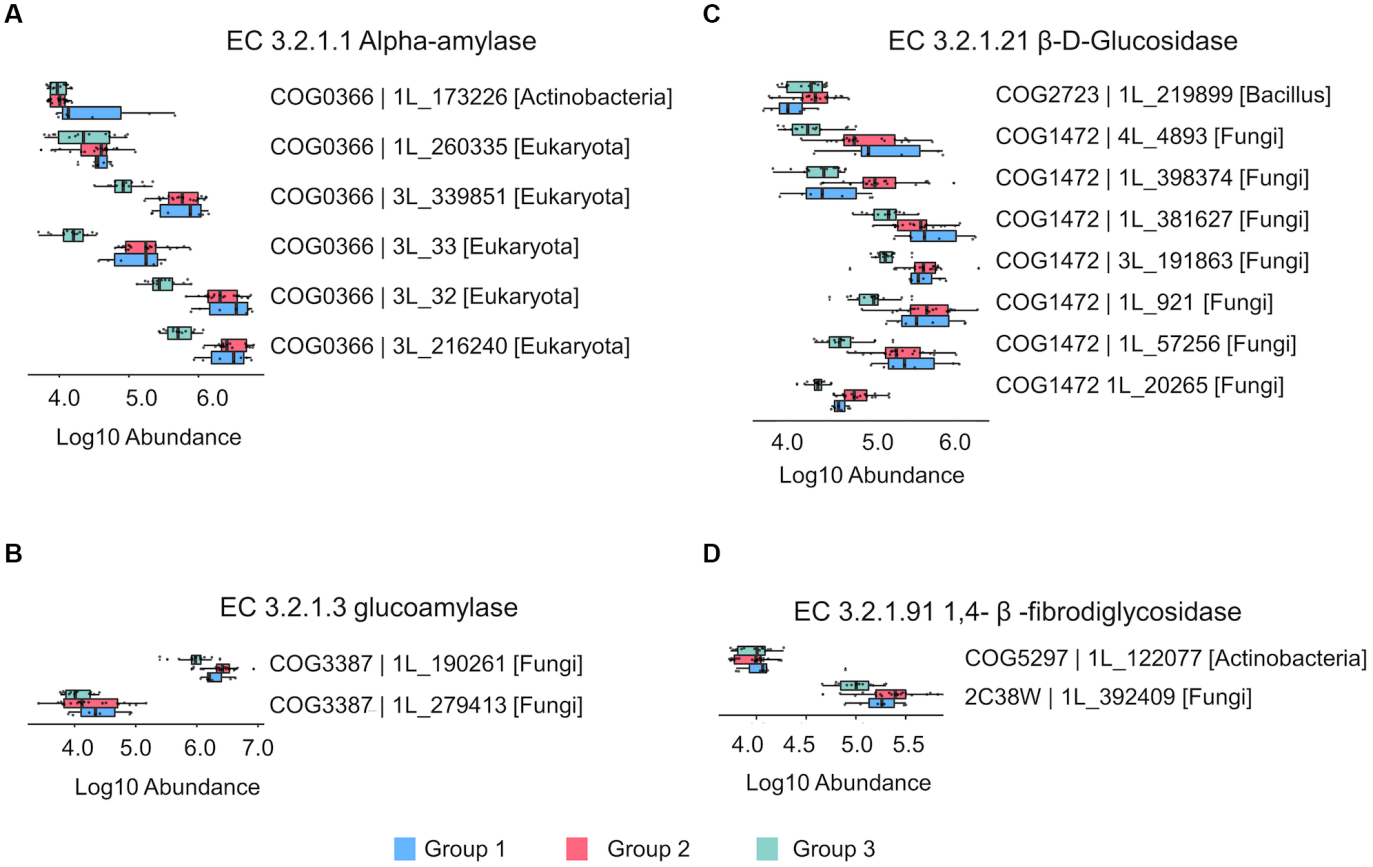
The bar plots showing the relative abundance of the key enzymes related to starch and cellulose hydrolysis process in the prepared *Daqu*. **(A)** Alpha-amylase (EC 3.2.1.1). **(B)** Glucoamylase (EC 3.2.1.3). **(C)** β-D-Glucosidase (EC 3.2.1.21). **(D)** 1,4-β-fibrodiglycosidase (EC 3.2.1.91). The boxes mark the first and third quantile and the lines inside the boxes mark the median; the whiskers extend from the ends of the inter-quartile range (IQR) to the furthest observations within the 1.5 times the IQR. Individual data points are overlaid as dots.

The abundances of the 18 enzymatic proteins in the three groups of prepared *Daqu* samples are shown in Figure 5 and Supplementary Data 8. The differences of abundances of the 18 enzymatic proteins among the 6 cycles are also shown in Supplementary Figure 7. Most of the proteins were less abundant in group 3 (cycles 5 and 6) compared to group 1 and group 2. PRM analysis was used to verify the enzymes. Fifteen of the 18 proteins were detected by PRM, and their relative quantities among the different cycles and groups by PRM maintained good agreement with the label-free DIA results (Supplementary Figure 8). In addition, we also analyzed the taxonomic source of the proteins. Most proteins (5) of α-amylase were from eukaryote, except 1 from Actinobacteria. Most proteins (7) of 1,4-β-fibrodiglycosidase and all the glucoamylase proteins (2) were from fungi, while 1 protein of 1,4-β-fibrodiglycosidase was from Bacilli. The proteins of β-D-glucosidase were from Actinobacteria (1) and fungi (1). Although the abundance of bacteria was higher than fungi in the prepared *Daqu* samples, the enzymes related to starch and cellulose hydrolysis were mainly from fungi.

## 4. Discussion

The brewing process of Moutai liquor is based on a traditional solid-state fermentation technique, where the yield and quality of produced liquor are highly correlated with the microorganisms in the starter of fermentation, i.e., the prepared *Daqu*. In this study, the microbial communities of prepared *Daqu* samples from 6 production cycles were investigated using a DIA-based label-free quantitative metaproteomic approach. It was found that the numbers of detected proteins and taxonomic compositions of the prepared *Daqu* samples were relatively stable across cycles, although the prepared *Daqu* samples were produced at different times of a year. This may be related to the microbial diversity of the prepared *Daqu*, and the production process of the prepared *Daqu*. The prepared *Daqu* for Moutai liquor is a mixture of different types of high-temperature *Daqu*, and hence is more stable than the individual high-temperature *Daqu* produced at different time of a year. As reported previously, the high diversity of microorganisms increases the tolerance to environmental disturbances during the fermentation process, thereby enhancing the stability of the microbial composition (27, 32). On the other hand, the consistency of numbers of detected proteins and peptides of the prepared *Daqu* samples from the same production cycle indicated that the DIA-based quantitative metaproteomics method has high reproducibility, demonstrating the great power of DIA-based metaproteomics for the characterization of microbiome in large sample cohorts.

Subsequently, we studied the microbial composition of the prepared *Daqu* samples, and found that the dominant genera were *Kroppenstedtia*, *Bacillus*, *Virgibacillus*, *Oceanobacillus*, *Staphylococcus*, and *Enterococcus*. Among them, *Bacillus*, *Virgibacillus*, *Oceanobacillus*, and *Staphylococcus* have been reported in a previous study of Moutai *Daqu* using 16S rRNA sequencing (5). In this study, it was found that the *Bacillus* genus had a relatively high abundance and the largest number (8) of detected species in the prepared *Daqu*. Moreover, *Bacillus* was the main genera that shaped the topology of the co-occurrence microbial networks in the prepared *Daqu* samples. *Bacillus* has strong viability under high temperature conditions, and thereby gradually became the dominant bacteria in the fermentation process. In addition, *Bacillus* is a significant source of protease and amylase with strong hydrolysis ability, and the hydrolysis products are important precursors of subsequent flavor compounds (10, 33). It has been reported that *Bacillus* can synthesize pyrazine, which contributes to baked, roasted and nutty flavors, creating the unique flavor of the final brewed liquor (27, 34, 35). Lactic acid produced by *Bacillus* can reduce the pH value of the fermentation, thereby inhibiting the growth of other microorganisms and promoting the production of acidic odors, which partially constitute the aroma components of liquor (27, 36, 37). It has also been reported that the saccharification ability of Moutai *Daqu* was positively correlated with *Bacillus* during fermentation (30). In this study, the abundance of *Bacillus* was the highest in group 3 of the production cycles among the three groups. However, metabolism of galactose, starch and sucrose was decreased in group 3. The key enzymes involving in the saccharification process was mainly from fungi instead of bacteria, and the abundances of these enzymes were lower in group 3 than those in group 1 and group 2.

Differential proteins and differential metabolic pathways were also found across the production cycles, and mainly included the metabolism of carbohydrates and amino acids. Among the 38 significantly differential pathways shown in Figure 4B, 7 were correlated with amino acids metabolism, an important process of microbial metabolism in *Daqu* (38). Amino acids are nitrogen sources for yeasts, and the lack of amino acids will directly affect the content of alcohols, esters, and other flavor substances (39). In group 3 of the prepared *Daqu*, metabolism of terpenoids and polyketides was significantly elevated. It has been reported that terpenoids generated during fermentation can make the aroma of Moutai liquor more elegant and delicate (40).

In summary, a DIA-based label-free quantitative metaproteomics approach was used to investigate the taxonomic composition and function profiles of prepared *Daqu* microbiota in different production cycles of Moutai liquor. The microbial compositions of the prepared *Daqu* samples were relatively stable across the 6 production cycles at different times of the year. *Bacillus* was the dominant bacteria in all samples. KEGG pathway analysis showed that the metabolism of carbohydrates and amino acids exhibited alteration in the prepared *Daqu* across the production cycles. These metabolic pathways were mainly related to the saccharification process in liquor fermentation and the synthesis of secondary metabolites to form the unique flavor and aroma of Moutai liquor. This study provides insights through metaproteomics to the control of yield and quality in the production of Chinese liquor. We note that these results are only based on metaproteomics and complementary results from others methods, such as metagenomics and metabolomics, are important to strengthen the findings in the future. In future research, specific enzymes produced by microorganisms during liquor fermentation can be investigated by combining targeted proteomics and metabolomics techniques, towards a thorough understanding of their abundances and activities across production cycles and the corresponding effects on liquor yield and special flavor compounds formation.

## Data availability statement

The original contributions presented in the study are publicly available. This data can be found here: ProteomeXchange via the iProX (41) partner repository with accession numbers PXD035791 or IPX0004814000 (https://www.iprox.cn/page/project.html?id=IPX0004814000).

## Author contributions

JzZ: conceptualization, investigation, methodology, formal analysis, and writing—original draft. YY: investigation, formal analysis, and writing—review and editing. MT: conceptualization, investigation, and resources. JxZ, VM, YL, ZF, and CS: investigation. BW: formal analysis. SY: resources. FY and LW: conceptualization, methodology, and resources. LQ: conceptualization, methodology, and writing—review and editing. All authors contributed to the article and approved the submitted version.

## Funding

This work was supported by the National Natural Science Foundation of China (NSFC, 22022401).

## Conflict of interest

JzZ, MT, FY, and LW are employed by Kweichow Moutai Group. CS is employed by Shanghai Omicsolution Co., Ltd.

The remaining authors declare that the research was conducted in the absence of any commercial or financial relationships that could be construed as a potential conflict of interest.

## Publisher’s note

All claims expressed in this article are solely those of the authors and do not necessarily represent those of their affiliated organizations, or those of the publisher, the editors and the reviewers. Any product that may be evaluated in this article, or claim that may be made by its manufacturer, is not guaranteed or endorsed by the publisher.

## References

[1] Xu Y Wang D Fan WL Mu XQ Chen J. Traditional Chinese biotechnology In: Tsao GT Ouyang P Chen J, editors. Advances in Biochemical Engineering-Biotechnology. Berlin, Heidelberg: Springer (2010). 189–233. 10.1007/10_2008_36 19888561

[2] Wang Y Cai W Wang W Shu N Zhang Z Hou Q. Analysis of microbial diversity and functional differences in different types of high-temperature Daqu. Food Sci Nutr. (2020) 9:1003–16. doi: 10.1002/fsn3.2068, PMID: 33598183 PMC7866569

[3] Yang J-G Dou X Han P-J Bai F-Y Zhou J Zhang S-Y. Microbial diversity in Daqu during production of Luzhou flavored liquor. J Am Soc Brew Chem. (2018) 75:136–44. doi: 10.1094/asbcj-2017-2879-01

[4] Yang J-G Dou X Ma Y-Y. Diversity and dynamic succession of microorganisms during Daqu preparation for Luzhou-flavour liquor using second-generation sequencing technology. J Inst Brew. (2018) 124:498–507. doi: 10.1002/jib.528

[5] Gan SH Yang F Sahu SK Luo RY Liao SL Wang HY. Deciphering the composition and functional profile of the microbial communities in Chinese Moutai liquor starters. Front Microbiol. (2019) 10:1540. doi: 10.3389/fmicb.2019.01540, PMID: 31333631 PMC6620787

[6] Li P Lin W Liu X Wang X Gan X Luo L. Effect of bioaugmented inoculation on microbiota dynamics during solid-state fermentation of Daqu starter using autochthonous of *Bacillus*, *Pediococcus*, Wickerhamomyces and Saccharomycopsis. Food Microbiol. (2017) 61:83–92. doi: 10.1016/j.fm.2016.09.004, PMID: 27697173

[7] Du H Wang X Zhang Y Xu Y. Exploring the impacts of raw materials and environments on the microbiota in Chinese Daqu starter. Int J Food Microbiol. (2019) 297:32–40. doi: 10.1016/j.ijfoodmicro.2019.02.020, PMID: 30878005

[8] Su Y Yang L Hui L Yuan-Yuan G Ming-Juan Z Chun-Hui X. Bacterial communities during the process of high-temperature Daqu production of roasted sesame-like flavour liquor. J Inst Brew. (2015) 121:440–8. doi: 10.1002/jib.235

[9] Liu P Zhang L Du X Zhao J Gao G Zhang X. Dynamic analysis of physicochemical and biochemical indices and microbial communities of light-flavor Daqu during storage. J Am Soc Brew Chem. (2019) 77:287–94. doi: 10.1080/03610470.2019.1629238

[10] Wang C-l Shi D-j Gong G-l. Microorganisms in Daqu: a starter culture of Chinese Maotai-flavor liquor. World J Microbiol Biotechnol. (2008) 24:2183–90. doi: 10.1007/s11274-008-9728-0

[11] Zheng XW Yan Z Nout MJ Boekhout T Han BZ Zwietering MH. Characterization of the microbial Community in Different Types of Daqu samples as revealed by 16s Rrna and 26s Rrna gene clone libraries. World J Microbiol Biotechnol. (2015) 31:199–208. doi: 10.1007/s11274-014-1776-z, PMID: 25395233

[12] Wang X Ban S Hu B Qiu S Zhou H. Bacterial diversity of Moutai-flavour Daqu based on high-throughput sequencing method. J Inst Brew. (2017) 123:138–43. doi: 10.1002/jib.391

[13] Wang X-D Qiu S-Y Li P Ban S-D. Analysis of microbial community structure in traditional and automated Moutai-flavor Daqu. J Am Soc Brew Chem. (2019) 77:140–6. doi: 10.1080/03610470.2019.1569886

[14] Hu Y Dun Y Li S Fu B Xiong X Peng N. Changes in microbial community during fermentation of high-temperature Daqu used in the production of Chinese ‘Baiyunbian’ liquor. J Inst Brew. (2017) 123:594–9. doi: 10.1002/jib.455

[15] Lagier JC Dubourg G Million M Cadoret F Bilen M Fenollar F. Culturing the human microbiota and Culturomics. Nat Rev Microbiol. (2018) 16:540–50. doi: 10.1038/s41579-018-0041-0 29937540

[16] Long S Yang Y Shen C Wang Y Deng A Qin Q. Metaproteomics characterizes human gut microbiome function in colorectal Cancer. npj Biofilms Microbiomes. (2020) 6:14. doi: 10.1038/s41522-020-0123-4, PMID: 32210237 PMC7093434

[17] Xiao M Yang J Feng Y Zhu Y Chai X Wang Y. Metaproteomic strategies and applications for gut microbial research. Appl Microbiol Biotechnol. (2017) 101:3077–88. doi: 10.1007/s00253-017-8215-7 28293710

[18] Liu M-K Tang Y-M Guo X-J Zhao K Penttinen P Tian X-H. Structural and functional changes in prokaryotic communities in artificial pit mud during Chinese Baijiu production. mSystems. (2020) 5:e00829–19. doi: 10.1128/mSystems.00829-19, PMID: 32209718 PMC7093824

[19] Wu C Deng J. Metaproteomic characterization of Daqu, a fermentation starter culture of Chinese liquor. J Proteomics Bioinform. (2016) 09:049–52. doi: 10.4172/jpb.1000388

[20] Wang B Wu Q Xu Y Sun B. Synergistic effect of multiple Saccharifying enzymes on alcoholic fermentation for Chinese Baijiu production. Appl Environ Microbiol. (2020) 86:e00013–20. doi: 10.1128/AEM 32060021 PMC7117924

[21] Aakko J Pietilä S Suomi T Mahmoudian M Toivonen R Kouvonen P. Data-independent acquisition mass spectrometry in Metaproteomics of gut microbiota—implementation and computational analysis. J Proteome Res. (2020) 19:432–6. doi: 10.1021/acs.jproteome.9b00606, PMID: 31755272

[22] Pietilä S Suomi T Elo LL. Introducing untargeted data-independent Acquisition for Metaproteomics of complex microbial samples. ISME Commun. (2022) 2:51. doi: 10.1038/s43705-022-00137-0 PMC9723653 37938742

[23] Muntel J Gandhi T Verbeke L Bernhardt OM Treiber T Bruderer R. Surpassing 10 000 identified and quantified proteins in a single run by optimizing current Lc-Ms instrumentation and data analysis strategy. Mol Omics. (2019) 15:348–60. doi: 10.1039/C9MO00082H, PMID: 31465043

[24] Gotti C Roux-Dalvai F Joly-Beauparlant C Mangnier L Leclercq M Droit A. Extensive and accurate benchmarking of Dia acquisition methods and software tools using a complex proteomic standard. J Proteome Res. (2021) 20:4801–14. doi: 10.1021/acs.jproteome.1c00490, PMID: 34472865

[25] Zhao J Yang Y Chen L Zheng J Lv X Li D. Quantitative Metaproteomics reveals composition and metabolism characteristics of microbial communities in Chinese liquor fermentation starters. Front Microbiol. (2023) 13:1098268. doi: 10.3389/fmicb.2022.1098268, PMID: 36699582 PMC9868298

[26] Zhao J Yang Y Xu H Zheng J Shen C Chen T. Data-independent acquisition boosts quantitative Metaproteomics for deep characterization of gut microbiota. npj Biofilms Microbiomes. (2023) 9:4. doi: 10.1038/s41522-023-00373-9, PMID: 36693863 PMC9873935

[27] Wang L. Research trends in Jiang-flavor Baijiu fermentation: from fermentation microecology to environmental ecology. J Food Sci. (2022) 87:1362–74. doi: 10.1111/1750-3841.16092, PMID: 35275413

[28] Bruderer R Bernhardt OM Gandhi T Miladinović SM Cheng L-Y Messner S. Extending the limits of quantitative proteome profiling with data-independent acquisition and application to acetaminophen-treated three-dimensional liver microtissues. Mol Cell Proteomics. (2015) 14:1400–10. doi: 10.1074/mcp.M114.044305, PMID: 25724911 PMC4424408

[29] Chong J Soufan O Li C Caraus I Li S Bourque G. Metaboanalyst 4.0: towards more transparent and integrative metabolomics analysis. Nucleic Acids Res. (2018) 46:W486–94. doi: 10.1093/nar/gky310, PMID: 29762782 PMC6030889

[30] Huerta-Cepas J Szklarczyk D Forslund K Cook H Heller D Walter MC. Eggnog 4.5: a hierarchical Orthology framework with improved functional annotations for eukaryotic, prokaryotic and viral sequences. Nucleic Acids Res. (2015) 44:D286–93. doi: 10.1093/nar/gkv1248, PMID: 26582926 PMC4702882

[31] Bastian M Heymann S. Gephi: an open source software for Explorating and manipulating networks. In *Third international AAAI Conference on Weblogs and Social Media* (2009). p. 361–362.

[32] Feng K Zhang Z Cai W Liu W Xu M Yin H. Biodiversity and species competition regulate the resilience of microbial biofilm community. Mol Ecol. (2017) 26:6170–82. doi: 10.1111/mec.14356, PMID: 28926148

[33] Beaumont M. Flavouring composition prepared by fermentation with Bacillus Spp. Int J Food Microbiol. (2002) 75:189–96. doi: 10.1016/s0168-1605(01)00706-1, PMID: 12036142

[34] Jin Y Li D Ai M Tang Q Huang J Ding X. Correlation between volatile profiles and microbial communities: a metabonomic approach to study Jiang-flavor liquor Daqu. Food Res Int. (2019) 121:422–32. doi: 10.1016/j.foodres.2019.03.021, PMID: 31108766

[35] Zhang H Zhang L Yu X Xu Y. The biosynthesis mechanism involving 2,3-Pentanedione and Aminoacetone describes the production of 2-Ethyl-3,5-Dimethylpyrazine and 2-Ethyl-3,6-Dimethylpyrazine by *Bacillus Subtilis*. J Agric Food Chem. (2020) 68:3558–67. doi: 10.1021/acs.jafc.9b07809, PMID: 32065523

[36] Yang F Chen L Liu Y Li J Wang L Chen J. Identification of microorganisms producing lactic acid during solid-state fermentation of Maotai flavour liquor. J Inst Brew. (2019) 125:171–7. doi: 10.1002/jib.537

[37] Yang F Zhang Q Liu Y Li J Wang L Chen J. Lactic acid biosynthesis pathways and important genes of *Lactobacillus Panis* L7 isolated from the Chinese liquor brewing microbiome. Food Biosci. (2020) 36:100627. doi: 10.1016/j.fbio.2020.100627

[38] Yang L Fan W Xu Y. Gc × Gc-Tof/Ms and Uplc-Q-Tof/Ms based untargeted metabolomics coupled with physicochemical properties to reveal the characteristics of different type Daqus for making soy sauce aroma and flavor type Baijiu. LWT Food Sci Technol. (2021) 146:111416. doi: 10.1016/j.lwt.2021.111416

[39] Wang YP Wei XQ Guo XW Xiao DG. Effect of the deletion of genes related to amino acid metabolism on the production of higher alcohols by *Saccharomyces Cerevisiae*. Bio Med Res Int. (2020) 2020:6802512. doi: 10.1155/2020/6802512, PMID: 33204707 PMC7665916

[40] Wang L Hu G Lei L Lin L Wang D Wu J. Identification and aroma impact of volatile Terpenes in Moutai liquor. Int J Food Prop. (2016) 19:1335–52. doi: 10.1080/10942912.2015.1064442

[41] Ma J Chen T Wu S Yang C Bai M Shu K. Iprox: an integrated proteome resource. Nucleic Acids Res. (2019) 47:D1211–7. doi: 10.1093/nar/gky869, PMID: 30252093 PMC6323926

