# Supplementary material for: Metaproteomics profiling of the microbial communities in fermentation starters (Daqu) during multi-round production of Chinese liquor
Source: Front Nutr. 2023 Jun 1;10:1139836. doi: 10.3389/fnut.2023.1139836 (PMC10267310; doi:10.3389/fnut.2023.1139836)
Supplement: Supplementary file 1 [file Data_Sheet_1.PDF]

Supplementary Material

1 Supplementary Figures and Tables

1.1 Supplementary Figures

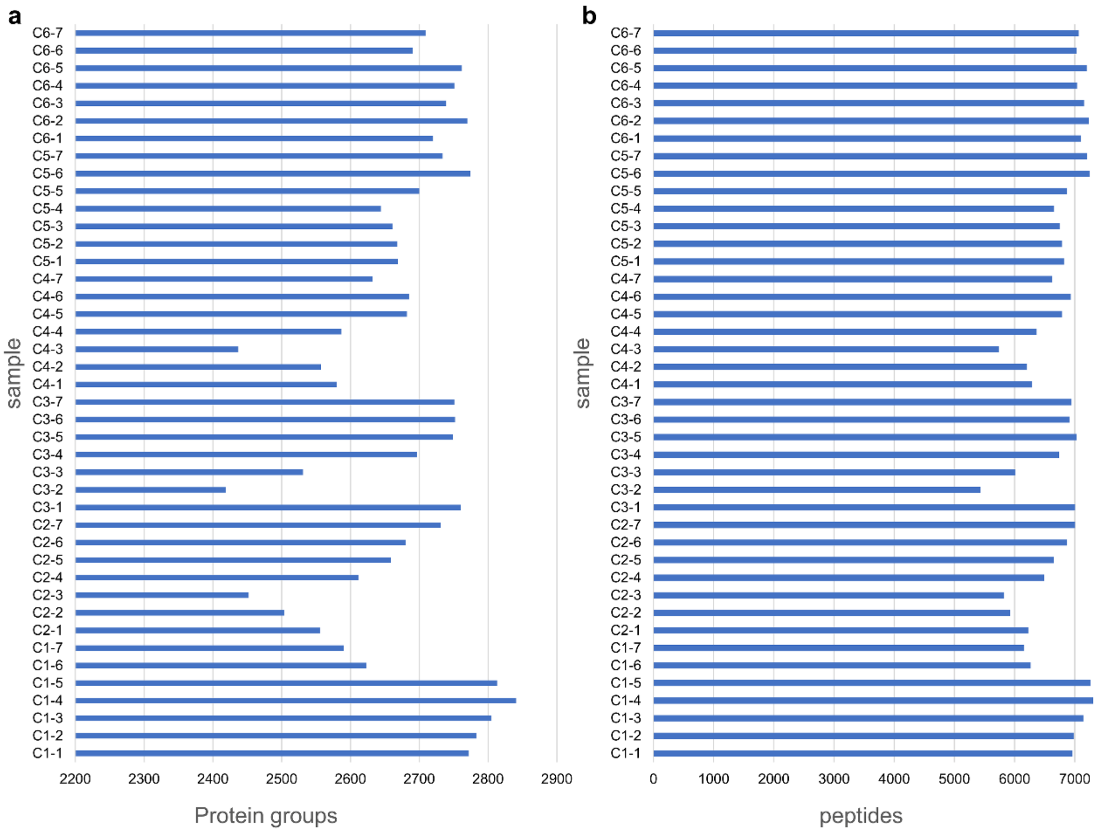

Supplementary Figure 1. Numbers of quantified (a) proteins and (b) peptides from the 42 prepared *Daqu* samples.

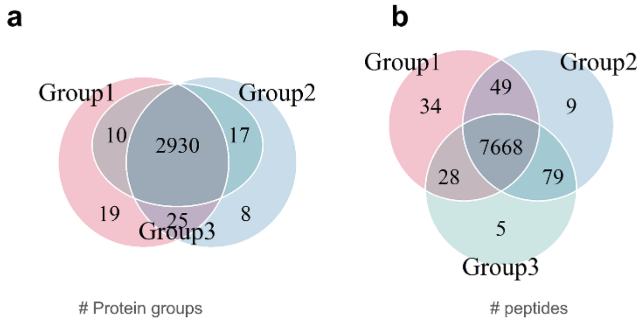

Supplementary Figure 2. The numbers of quantified (a) proteins and (b) peptides accumulated from the three groups of samples.

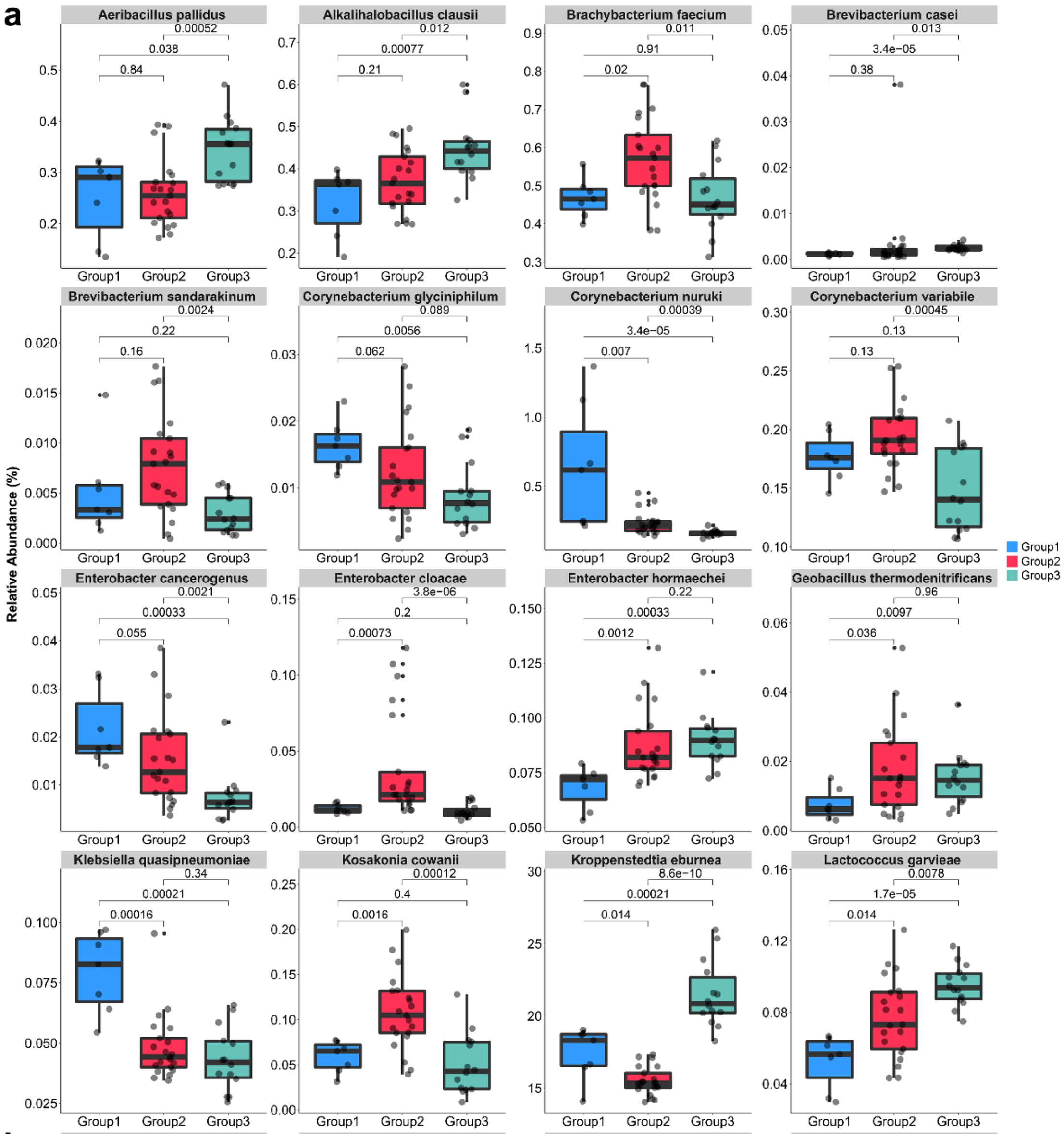
a
Relative Abundance (%)
Aeribacillus pallidus
Alkalihalobacillus clausii
Brachybacterium faecium
Brevibacterium casei
Brevibacterium sandarakinum
Corynebacterium glyciniphilum
Corynebacterium nuruki
Corynebacterium variabile
Enterobacter cancerogenus
Enterobacter cloacae
Enterobacter hormaechei
Geobacillus thermodenitrificans
Klebsiella quasipneumoniae
Kosakonia cowanii
Kroppenstedtia eburnea
Lactococcus garvieae
Group1
Group2
Group3

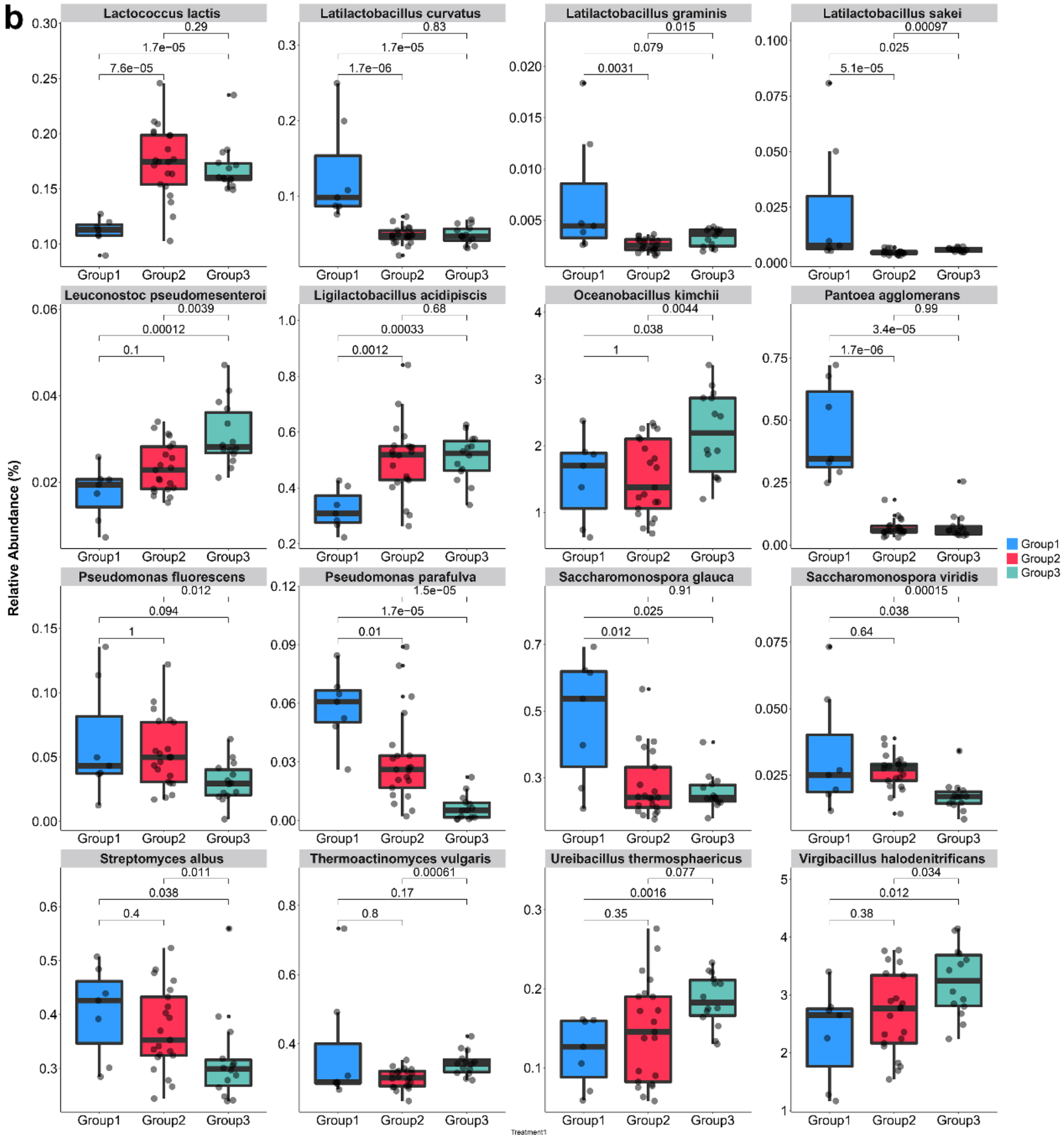

Supplementary Figure 3. The box plots showing the abundances of the differential taxa among the 3 groups (Krustal-Wails test p-value < 0.05). The boxes mark the first and third quantile and the lines inside the boxes mark the median; the whiskers extend from the ends of the inter-quartile range (IQR) to the furthest observations within the 1.5 times the IQR. Individual data points are overlaid as dots.

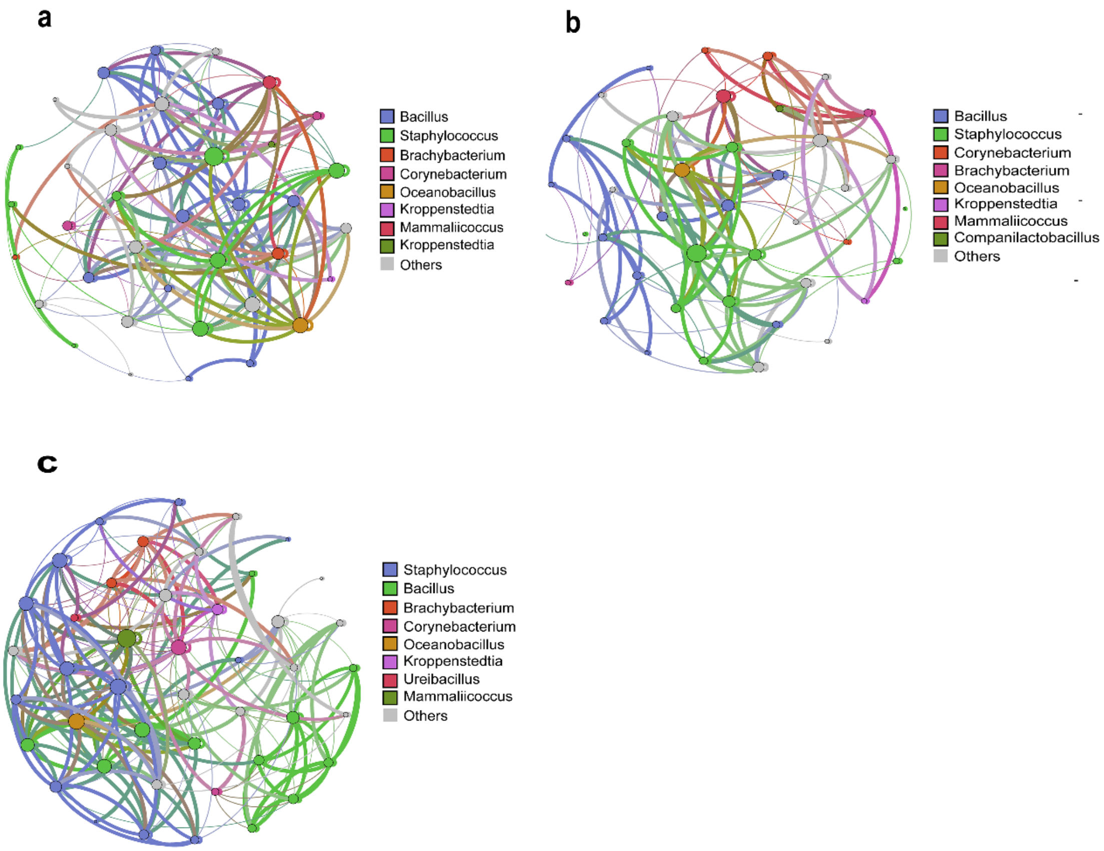

Supplementary Figure 4. Co-occurrence networks of the *Daqu* microbiota from cycle 2 (a), cycle 4 (b), and cycle 5 (c). Genera with the relative abundance > 0.1% and appeared in at least 80% of the samples are shown as nodes. A connection stands for significant correlation (p-value < 0.05). Size of each node is proportional to the number of connections, the nodes are colored by genera occupancy, and the thickness of edge is proportional to the absolute value of Spearman correlation coefficients.

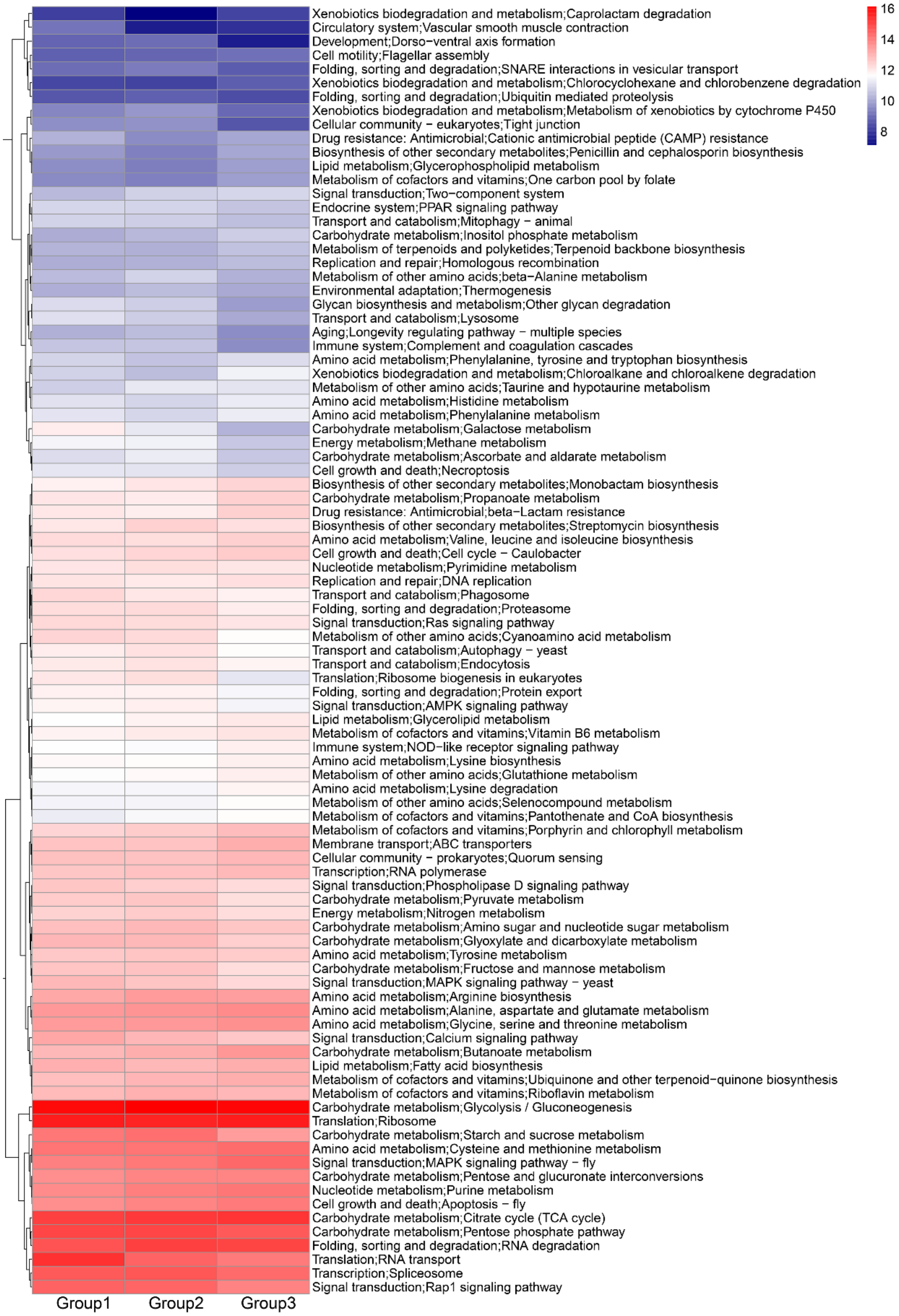

Supplementary Figure 5. Heatmap showing relative abundance of the differential pathways among the 3 groups (Krustal-Wails test p-value < 0.05).

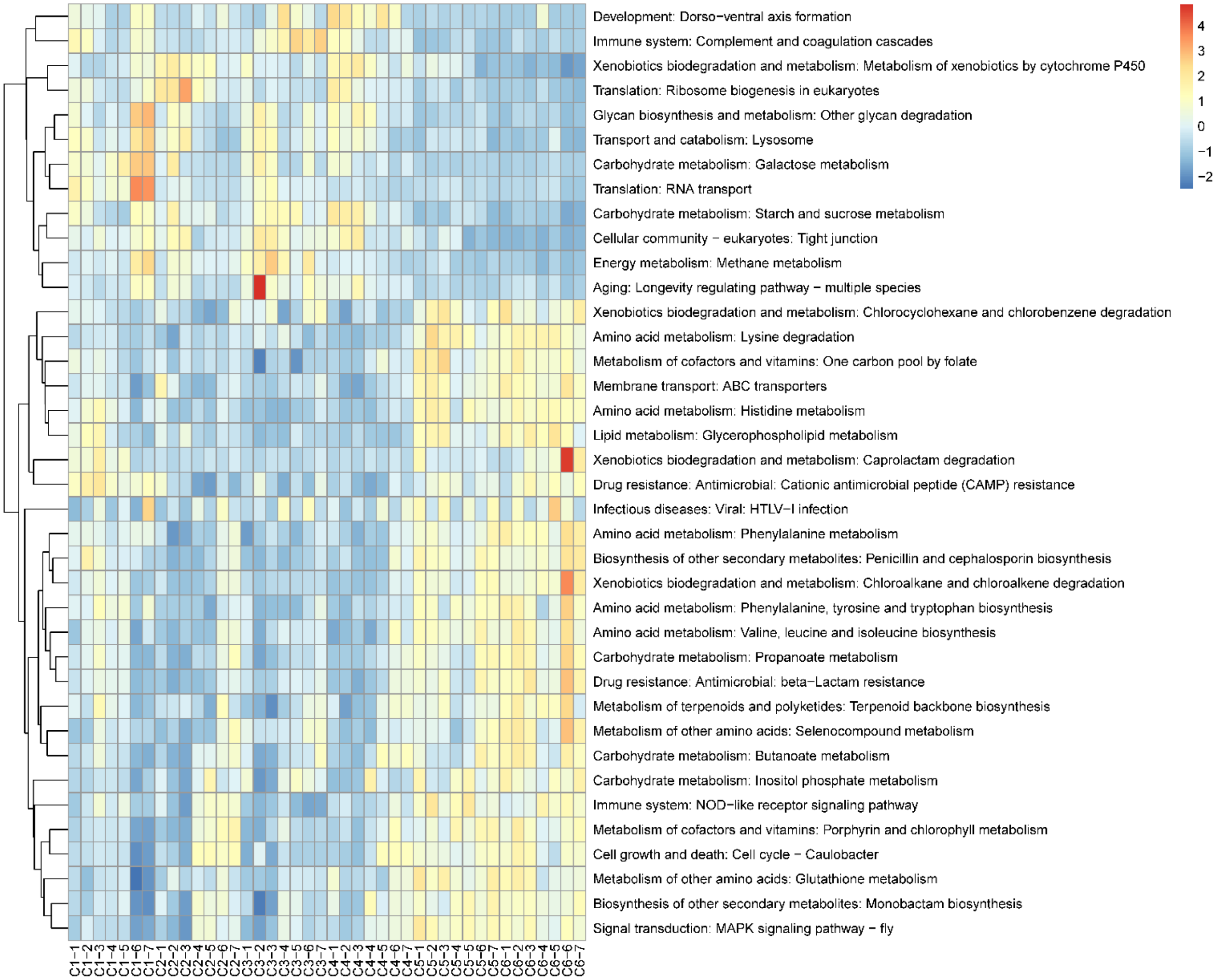

Supplementary Figure 6. Heatmap showing relative abundance of the differential pathways (fold change > 1.25 or < 0.5, and p-value < 0.05 by pairwise Wilcoxon test) among all the samples. Abundances are normalized to z-scores, which are in the units of standard deviation from the mean.

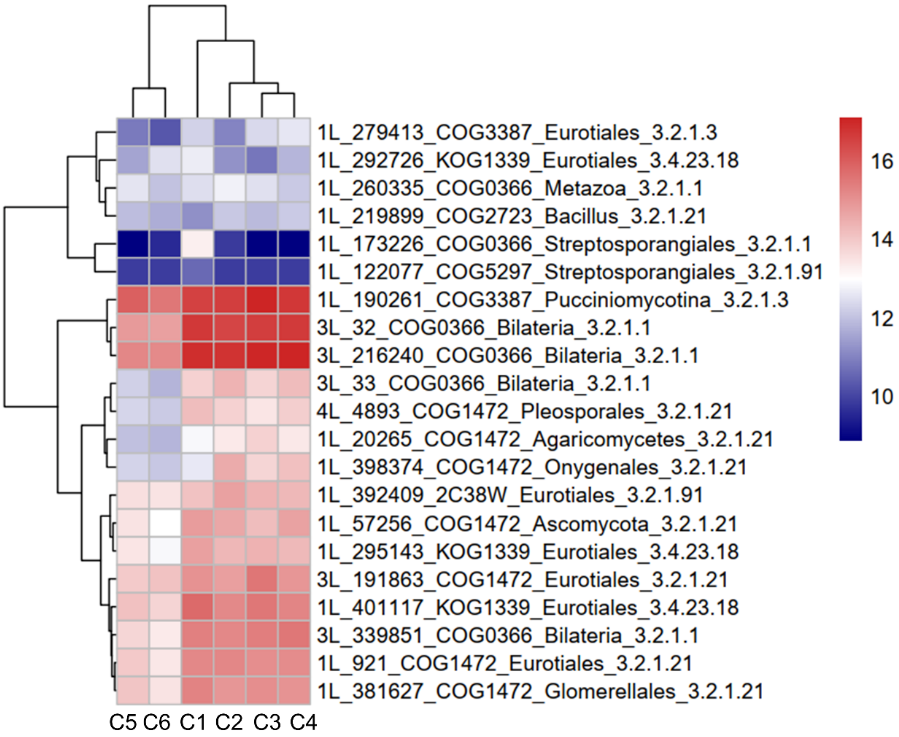

Supplementary Figure 7. Heatmap showing the relative abundance of the key enzymes related to starch and cellulose hydrolysis process in prepared *Daqu* among different cycles.

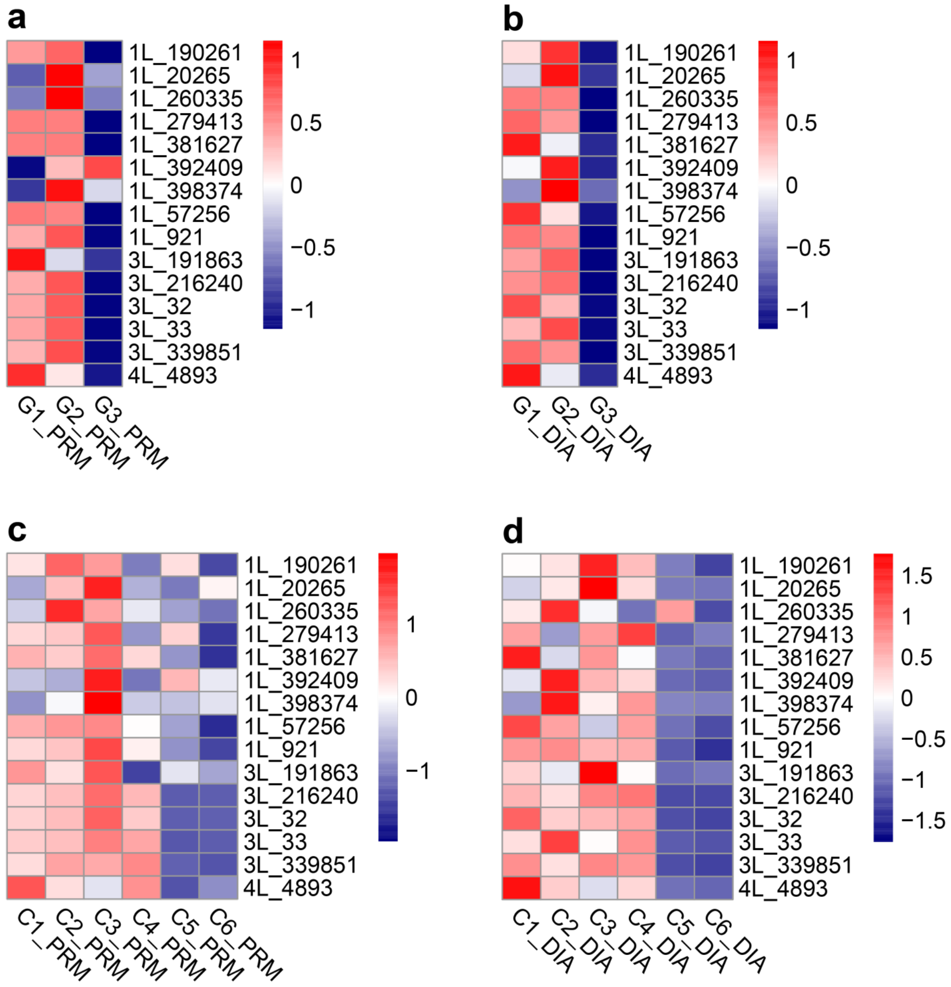

Supplementary Figure 8. Heatmaps showing the relative abundance (z-score) of 15 key enzymes related to starch and cellulose hydrolysis process in the prepared *Daqu* among the 3 groups using parallel reaction monitoring (PRM) (a) and label-free data-independent acquisition (DIA) (b), and among the 6 production cycles using PRM (c) and label-free DIA (d).

1.2 Supplementary Tables

Supplementary Table 1. Information of the collected prepared *Daqu* samples.

Sample name	Sampling date	Sample name	Sampling date
C1-1	2020.12.8	C4-1	2021.4.25
C1-2	2020.12.8	C4-2	2021.4.25
C1-3	2020.12.8	C4-3	2021.4.25
C1-4	2020.12.8	C4-4	2021.4.25
C1-5	2020.12.8	C4-5	2021.4.25
C1-6	2020.12.8	C4-6	2021.4.25
C1-7	2020.12.8	C4-7	2021.4.25
C2-1	2021.1.13	C5-1	2021.6.3
C2-2	2021.1.13	C5-2	2021.6.3
C2-3	2021.1.13	C5-3	2021.6.3
C2-4	2021.1.13	C5-4	2021.6.3
C2-5	2021.1.13	C5-5	2021.6.3
C2-6	2021.1.13	C5-6	2021.6.3
C2-7	2021.1.13	C5-7	2021.6.3
C3-1	2021.3.15	C6-1	2021.7.13
C3-2	2021.3.15	C6-2	2021.7.13
C3-3	2021.3.15	C6-3	2021.7.13
C3-4	2021.3.15	C6-4	2021.7.13
C3-5	2021.3.15	C6-5	2021.7.13
C3-6	2021.3.15	C6-6	2021.7.13
C3-7	2021.3.15	C6-7	2021.7.13

C1: cycle 1; the rest are in the same manner.

Supplementary Table 2. Chromatography gradient.

Time	B%
0:00	2%
4:00	5%
45:00	18%
50:00	22%
53:00	32%
56:00	95%
60:00	95%

Supplementary Table 3. DIA variable window settings.

Center	Width	Center	Width
368.5	38	669.5	10
399	25	678.5	10
418	15	688	11
432	15	697.5	10
444.5	12	706.5	10
455	11	716	11
464.5	10	726	11
473.5	10	736.5	12
482.5	10	747.5	12
491	9	758.5	12
499.5	10	769.5	12
508	9	780.5	12
516.5	10	792	13
525	9	804.5	14
533	9	818	15
541.5	10	832.5	16
550	9	848.5	18
558	9	866	19
566.5	10	885.5	22
575.5	10	907	23
584	9	929	23
592	9	951.5	24
600.5	10	974.5	24
609	9	998	25
617.5	10	1024.5	30
626	9	1056	35
634.5	10	1092.5	40
643.5	10	1139	55
652	9	1208	85
660.5	10	1375	251
